# Expression and Characterization of Monomeric Recombinant Isocitrate Dehydrogenases from *Corynebacterium glutamicum* and *Azotobacter vinelandii* for NADPH Regeneration

**DOI:** 10.3390/ijms232315318

**Published:** 2022-12-05

**Authors:** Hun-Dong Lee, Su-Kyoung Yoo, Ho-Seok Yoo, Chul-Ho Yun, Geun-Joong Kim

**Affiliations:** 1 Department of Biological Sciences and Research Center of Ecomimetics, College of Natural Sciences, Chonnam National University, Yongbong-ro, Buk-gu, Gwangju 61186, Republic of Korea; 2 School of Biological Sciences and Technology, Chonnam National University, Yongbong-ro, Buk-gu, Gwangju 61186, Republic of Korea

**Keywords:** monomeric isocitrate dehydrogenase, recombinant protein, hyper-level expression, NADPH-regeneration system

## Abstract

The enzymatic transformation of various chemicals, especially using NADPH-dependent hydroxylase, into more soluble and/or high value-added products has steadily garnered increasing attention. However, the industrial application of these NADPH-dependent hydroxylases has been limited due to the high cost of the cofactor NADPH. As an alternative, enzymatic NADPH-regeneration systems have been developed and are frequently used in various fields. Here, we expressed and compared two recombinant isocitrate dehydrogenases (IDHs) from *Corynebacterium glutamicum* and *Azotobacter vinelandii* in *Escherichia coli*. Both enzymes were hyper-expressed in the soluble fraction of *E. coli* and were single-step purified to apparent homogeneity with yields of more than 850 mg/L. These enzymes also functioned well when paired with NADPH consumption systems. Specifically, NADPH was regenerated from NADP^+^ when an NADPH-consuming cytochrome P450 BM3 from *Bacillus megaterium* was incorporated. Therefore, both enzymes could be used as alternatives to the commonly used regeneration system for NADPH. These enzymes also have promising potential as genetic fusion partners with NADPH-dependent enzymes due to the monomeric nature of their quaternary structure, thereby resulting in self-sufficient biocatalysts via NADPH regeneration in a single polypeptide with NADPH-dependent activity.

## 1. Introduction

Since the discovery of the high selectivity and functions of enzymes in mild reaction conditions, transformation processes using enzymes have become an attractive alternative to chemical catalysis [1,2]. Among these enzymes, hydroxylases including cytochrome P450 BM3, which uses nicotinamide adenine dinucleotide phosphate (NADPH) as a cofactor, have great application potential in various fields such as drug discovery, bioremediation, detoxification, and chemistry [3]. These enzymes also play critical roles in drug metabolism, de novo synthesis, radical quenching, and immune defense in vivo. Therefore, studies on gene mining, enzyme characterization, and process developments are steadily gaining traction. However, the industrial application of NADPH-dependent enzyme requires a large amount of NADPH for the reaction to proceed, and NADPH is naturally oxidized under reaction conditions, making this process extremely costly [4]. To solve these limitations, NADPH regeneration systems have been developed, and are commonly coupled with enzymatic, electrochemical, and optical processes. Among these systems, enzyme-coupled NADPH regeneration systems have been widely applied [5]. These systems rely on various dehydrogenases that catalyze the reduction of the cofactor NADP^+^ to NADPH by oxidizing a specific substrate [6]. Typically, these dehydrogenases include glucose dehydrogenase (GDH), formate dehydrogenase (FDH), and glucose-6-phosphate dehydrogenase (G6PDH) [7,8], and have been successfully coupled with NADPH-dependent enzymes [7,8,9,10]. These systems have been further improved by co-immobilization and co-assembly of NADPH-dependent enzymes with regeneration enzymes to improve the stability and activity of the two enzymes, as well as the electron transfer efficiency between them [11,12,13]. However, few attempts have been made to develop more effective strategies to enhance electron transfer via genetic fusion for the generation of fusion proteins [14,15]. This is likely because all enzymes used in NADPH regeneration systems have multimeric structures such as dimers and tetramers. In turn, these structural characteristics often result in low expression levels and protein aggregation [16], especially when genetically fused with other proteins. Therefore, there is a need to discover novel enzymes with monomer structures that can replace the multimeric enzymes used in NADPH-regeneration systems.

NADP^+^-dependent isocitrate dehydrogenase (IDH, E.C. 1.1.1.42) catalyzes the oxidative decarboxylation of isocitrate to α-ketoglutarate and CO_2_ with simultaneous reduction of NADP^+^ to NADPH in the tricarboxylic acid (TCA) cycle. Generally, IDHs have a molecular weight of 40–60 kDa and form a dimer. However, some bacteria such as *Corynebacterium glutamicum*, *Azotobacter vinelandii*, and *Vibrio parahaemolyticus* are known to possess monomeric IDH with a calculated molecular mass of approximately 80 kDa [17,18,19]. Previous studies on IDH have mainly focused on the cancer-related functions of eukaryotic IDH [20]. However, very few studies have analyzed the NADPH regeneration capacity of these enzymes [21] and, to the best of our knowledge, no previous studies have attempted the functional genetic fusion of monomeric IDH with NADPH-dependent enzymes.

In this study, IDHs from *C. glutamicum* and *A. vinelandii* (CgIDH and AvIDH, respectively) were functionally overexpressed in *E. coli* to evaluate the applicability of monomeric IDH as a component of an NADPH regeneration system. The resulting recombinant proteins were purified to apparent homogeneity in a single step using Ni-NTA chromatography, after which their physicochemical properties were compared in terms of their specific activity, kinetic constants, and quaternary structure. Additionally, the performance of our proposed monomeric IDH-coupled regeneration system for NADPH was compared to those of G6PDH from *Leuconostoc pseudomesenteroids* (LmG6PDH) by using cytochrome P450 from *Bacillus megaterium* (BM3) as a NADPH-dependent (consuming) enzyme. Finally, we discussed the plausibility and applicability of the genetic fusion of monomeric IDH with an NADPH-dependent enzyme.

## 2. Results

### 2.1. Expression and Purification of CgIDH and AvIDH

**Figure 1:**
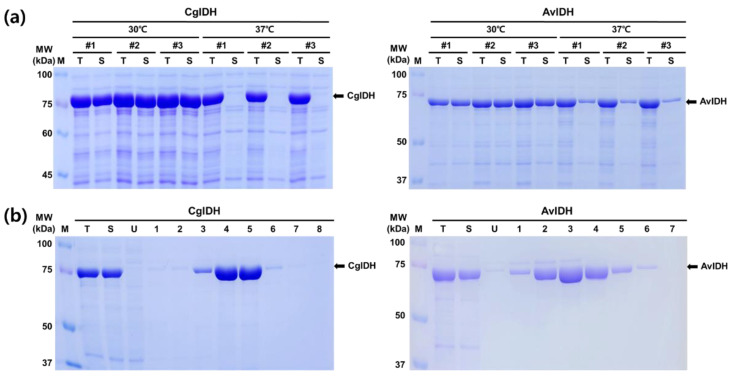
SDS-PAGE analyses of the expressed and purified recombinant IDHs. Aliquots of all protein samples were analyzed on a 10% PAGE gel under denaturing conditions. (**a**) The expression profiles and solubilities of the recombinant CgIDH and AvIDH were analyzed under two different induction conditions at 30 and 37 °C. (**b**) SDS-PAGE analyses of the elution profiles of recombinant CgIDH and AvIDH from the Ni-NTA column. M, molecular weight size marker; T, total protein fraction; S, soluble protein fraction; U, unbinding protein fraction; 1–8, eluted protein fractions. The arrows indicate the bands corresponding to the recombinant CgIDH and AvIDH.

The two amplified genes encoding IDH from the corresponding genomic DNA were subcloned into the pET24a expression vector to generate the recombinant plasmids pET24a-CgIDH and pET24a-AvIDH. Considering the results of related research for recombinant protein expression, a 6xHis-tag was incorporated into the C-terminal region of IDH to minimize the effect on enzyme activity [22]. The transformed cells often exhibited growth retardation at 37 °C compared to the control cells with an empty vector. Upon induction with IPTG, almost all of the recombinant proteins of CgIDH were expressed in the insoluble fraction at 37 °C (Figure 1a). Approximately 15–20% of recombinant AvIDH was only detected in the soluble fraction under the same conditions. The changes in induction conditions did not significantly improve the expression ratio in the soluble fraction. In contrast, when the protein expression was induced at 30 °C, both proteins were mainly expressed (>85%) in the soluble fraction (Figure 1a). These results were consistently observed in repeated experiments. Further lowering the induction temperature to 25 °C decreased protein expression compared to 30 °C. In fact, as observed in Figure 1, the expressed protein AvIDH exhibited a lower molecular mass than expected. The theoretically calculated molecular mass of AvIDH is approximately 81.3 kDa. However, the corresponding band on the SDS-PAGE gel was determined to be roughly 72–74 kDa and showed the same size in all induction experiments under varying conditions. This result was unexpected and the reason for this outcome remain unclear. The alignment of primary structures between the two enzymes CgIDH and AvIDH resulted in a high sequence identity (61.6%) and revealed more homology when conservative substitution was considered (Figure S1). Next, we sought to preliminarily purify the recombinant protein AvIDH and further attempt to assess its enzymatic activity. As described below, the purified AvIDH with reduced size was functional, and stable, and its properties were comparable to that of the purified enzyme from the related strains [17,23,24,25]. Based on these observations, we purified the two recombinant proteins CgIDH and AvIDH to apparent homogeneity, after which their biochemical properties were compared in detail under the same conditions.

As shown in Figure 1b, the two recombinant proteins CgIDH and AvIDH were efficiently purified to apparent homogeneity through a single-step method using Ni-NTA chromatography. For the elution profiles, CgIDH and AvIDH were eluted at imidazole concentrations of 100–125 mM and 50–100 mM, respectively. These results also confirmed the presence of the His-tags at the C-terminus of AvIDH despite its reduced size. SDS-PAGE analysis of the purified proteins also showed that the molecular masses of the two recombinant proteins were consistent with those observed in the crude extract. There were no bands corresponding to the calculated molecular mass of AvIDH (81.3 kDa) in the eluted fraction. Further degradation and/or aggregation of the purified AvIDH was not observed after 7 days of storage at 4 °C. These results partly demonstrated that the proteolytic degradation of vulnerable IDH was not the main cause of size reduction.

### 2.2. Quaternary Structure Analyses of CgIDH and AvIDH

**Figure 2:**
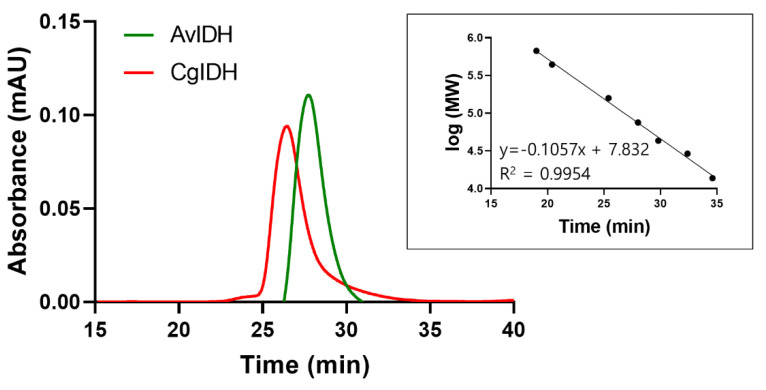
Size exclusion chromatography analyses of the purified CgIDH and AvIDH. The purified proteins ranging from 75 to 100 μg were analyzed on a Superdex 200 Increase 10/300 GL column. The red and green lines indicate CgIDH and AvIDH, respectively. The insert figure shows the calibration curve with a molar mass standard: thyroglobulin (669 kDa), ferritin (440 kDa), aldolase (158 kDa), conalbumin (75 kDa), ovalbumin (44 kDa) carbonic anhydrase (29 kDa), and ribonuclease A (13.7 kDa). The shift in elution time (<0.2 min) among the three repetitions was negligible.

Based on the estimated molecular masses of the two recombinant proteins, the relative mobility and quaternary structure were determined via SEC. As shown in Figure 2, the elution profiles of CgIDH and AvIDH exhibited single and symmetric peaks corresponding to molecular masses of approximately 80 and 75 kDa, respectively. These results were consistent with the relative size and mobility of two proteins on the SDS-PAGE gel. Therefore, these results also demonstrated that CgIDH and AvIDH existed as a monomer in solution, which was also consistent with previous reports that showed the identical results using the same or related enzymes [17,18,25]. These characteristics were maintained under various salt (NaCl) and imidazole concentrations and after prolonged storage at 4 °C for 30 days.

### 2.3. Kinetic Parameters and Biochemical Properties of CgIDH and AvIDH

As previously reported, the two recombinant proteins CgIDH and AvIDH showed a completely dependent activity toward NADP^+^ as a cofactor. In our experiments, there was no detectable activity toward another cofactor NAD^+^. The specific activity (U/mg) of CgIDH and AvIDH toward the substrate DL-isocitrate were determined to be 135.0 ± 4.8 and 79.1 ± 5.0 U/mg by using NADP^+^ as a cofactor, respectively. We also determined the kinetic parameters for CgIDH and AvIDH, which are summarized in Table 1. The *K*_m_ values of CgIDH for NADP^+^ and isocitrate were calculated to be 10.3 and 15.0 μM, respectively, which were 2.6- to 3-fold higher than those from an earlier study on the same enzyme purified from wild-type *C. glutamicum* [25]. The catalytic efficiency (*k*_cat_/*K*_m_) of CgIDH for NADP^+^ and isocitrate were 4.3 and 3.3 μM^−1^s^−1^, respectively, which were 5.1- to 5.3-fold lower than those from the aforementioned report. The *K*_m_ values of AvIDH for NADP^+^ and isocitrate were determined to be 5.9 and 13.9 μM, respectively, which were similar to those of previously reported enzyme with an N-terminal His-tag [26], and the catalytic efficiency of AvIDH for NADP^+^ and isocitrate were 6.6 and 3.2 μM^−1^ s^−1^, respectively, which were approximately 2.4- and 2.9-fold lower than those of a previously reported enzyme (15.9 and 9.3 μM^−1^s^−1^).

We also investigated other biochemical properties of the two enzymes and summarized them in Table 1. Regarding the optimum reaction temperature, the activities of CgIDH and AvIDH reached their maximum at 40 °C, which was in line with the optimal temperatures reported in previous studies that evaluated monomeric IDHs [23,24]. The specific activities of the two enzymes at temperatures above 40 °C decreased sharply due to their low thermostability. In contrast, CgIDH and AvIDH showed a broad range of optimal pH values from 7 to 9, which were also similar to those of other monomeric IDHs [23,24]. Additionally, the effects of different metal ion and salt concentrations on enzyme activity were also investigated (Table 2 and Figure S2).

As summarized in Table 2, the activities of CgIDH and AvIDH were highly influenced by the presence of metal ions and their highest activity was achieved in reaction buffer supplemented with Mn^2+^. The other metal ions, namely Ca^2+^, Cu^2+^, and Ni^2+^, severely inhibited the activities of both enzymes. Typical salts such as NaCl did not distinctly affect the activity of CgIDH even at concentrations as high as 500 mM. In contrast, the activity of AvIDH decreased gradually with higher NaCl concentrations, and was even more severely inhibited by KPi (Figure S2). This was likely due to the chelating effect of KPi on metal ions. Taken together, our finding indicated that although the activity of the two enzymes at relatively high temperatures could be further improved, both enzymes could produce sufficient amounts of NADPH for the cofactor-dependent enzyme under normal reaction conditions. Therefore, we next sought to evaluate the function of the two enzymes in a coupling system using NADPH-dependent P450 BM3 as a proof of concept.

### 2.4. Regeneration of NADPH Using Monomeric CgIDH and AvIDH

**Figure 3:**
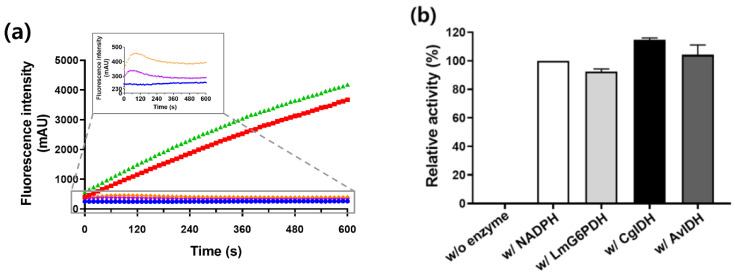
Coupling reaction of P450 BM3 with IDH for NADPH regeneration. The coupling reactions of BM3 with IDHs were carried out as described in the Materials and Methods. (**a**) The increase of NADPH fluorescence in the reaction mixture was monitored using a fluorometer. The negative control without IDH, the reaction with only CgIDH, the reaction with only AvIDH, the BM3 reaction coupled with CgIDH, and the BM3 reaction coupled with AvIDH are indicated in Blue, red, green, orange, and purple, respectively. The insert figure shows an initial increase followed by a constant level of NADPH in the coupled reaction. (**b**) Aliquots of the coupled reaction samples of (**a**) were taken after 10 min of reaction and treated with cold-methanol (1:1), then analyzed by high performance liquid chromatography. As a positive control, typically used LmG6PDH as a NADPH regeneration enzyme was assayed under the same conditions. The BM3 reaction with NADPH, instead of NADP^+^, was also used as a control. The reaction without BM3 was used as a negative control. With CgIDH and AvIDH indicates the coupling reaction with CgIDH and AvIDH, respectively.

To investigate the NADPH regeneration ability of CgIDH and AvIDH, the NADPH-dependent cytochrome P450 BM3 M10 was arbitrarily selected as a model enzyme [27]. As previously reported, BM3 M10 could catalyze omeprazole (OMP) into 5-hydroxyomeprazole (5-OH-OMP) by hydroxylation in an NADPH-dependent manner [28]. First, we measured the profile of NADPH fluorescence in the coupling reaction of BM3 with CgIDH or AvIDH (Figure 3a). No change in fluorescence was observed in the negative control group (without IDH). In the positive control group (without BM3), the fluorescence of NADPH, which was reduced by IDH from NADP^+^, was found to increase. As expected, the NADPH fluorescence from the coupling reaction of BM3 with CgIDH or AvIDH was maintained at a certain level after a slight increase in the fluorescence at the beginning of the reaction.

These results indicated that cofactor recycling occurred between NADPH-consuming BM3 and NADPH-supplying IDH. Next, we performed HPLC analysis to confirm that the 5-OH-OMP was indeed produced by the activity of BM3 in the coupling reaction (Figure 3b). Upon comparing the reaction solution of BM3 with NADPH as a positive control, BM3 coupled with CgIDH or AvIDH showed similar activity to the positive control. Intriguingly, the relative activities of BM3 in the two coupling systems were higher than that of another control group that used LmG6PDH as a typical NADPH-regenerator under the same reaction conditions. These results suggested that monomeric IDH could be used as an alternative enzyme in the NADPH-regeneration system.

## 3. Discussion

Several NADPH-regeneration systems are being actively developed for practical applications involving NADPH-dependent enzymes for the production of various high-value-added substances [5,6]. Among them, a method using an enzyme that reduces NADP^+^ to NADPH via oxidation of a specific substrate has been primarily considered as a coupled enzyme for NADPH regeneration. Although several plausible candidates for this purpose have been proposed, almost all related studies have mainly focused on the simple mixing of independently expressed NADPH-consuming and regenerating enzymes in a separated state. Ideally, the direct fusion between two enzymes in a single polypeptide is promising, as this would enhance the electron channeling between the two enzymes [14,15]. However, very few studies have attempted to genetically fuse NADPH-consuming enzymes with regeneration enzymes. This is likely because most enzymes used in NADPH-regeneration systems are multimers, which causes difficulties in protein expression due to abnormal folding and aggregation [16,29]. In this study, we suggested two monomeric IDHs as alternative enzymes for NADPH regeneration based on previously reported results for the purified wild-type enzymes from *C. glutamicum* [25] and *A. vinelandii* [18]. As described in the Results section, the two recombinant enzymes with the C-terminal His-tag were functionally overexpressed in *E. coli* mainly in soluble form. The expression levels of the two enzymes were determined to be over 35% of the total proteins and were easily purified to apparent homogeneity in a single step using metal-binding affinity chromatography. The production yield of CgIDH and AvIDH were at least 0.85 g/L in typical batch culture conditions and could be further increased through high-cell density cultivation and process optimization [30]. Additionally, although the molecular weight of AvIDH was lower than expected and the reasons for this phenomenon are yet to be elucidated, our finding provided some information regarding the characteristics of the two recombinants IDHs. Moreover, AvIDH remained active under various salt concentrations and is therefore expected to produce a sufficient amount of NADPH for NADPH-dependent enzymes via coupling reactions. Furthermore, although the amounts of CgIDH and AvIDH were lower than that of BM3 in the coupling reaction, BM3 activity was well driven by the activity of IDH, which reduced NADP^+^ to NADPH (Figure 3). Ongoing efforts are being made to further validate the performance of coupling reactions at a large scale.

As discussed above, the monomeric form of IDHs reported herein was expected to possess a stronger fusion ability than that of multimeric enzymes. As a preliminary experiment, direct fusion of NADPH-consuming BM3 M10 with NADPH-supplying IDH was attempted at the gene level. To this end, two proteins were genetically linked by a specific linker (EAAAK)_4_ and then expressed as described in the experimental section. As expected, BM3-IDHs (BM3-CgIDH and BM3-AvIDH) were expressed in the soluble fractions despite their large molecular weight of approximately 200 kDa (Figure S3a). The fusion proteins were then partially purified by metal-affinity chromatography using His-tag at their C-terminal regions. As shown in Figure S3b, both fusion proteins were functional and produced 5-OH-OMP in the reaction solution containing OMP and NADP^+^. These results provided evidence that NADPH-consuming BM3 and NADPH-supplying IDH maintained their activities in the fusion state, thereby showing the fusion ability of both CgIDH and AvIDH. However, BM3-IDHs showed lower production yield and enzyme activity than those of independently expressed BM3 and IDH. Therefore, additional efforts are being made to develop fusion proteins with improved properties by changing the gene order and linkers or through protein engineering such as random mutagenesis and/or DNA shuffling [29,31]. Chemical coupling and immobilization of the resulting proteins could also substantially improve catalytic performance [11,12,13]. Therefore, it is important to verify whether the monomeric IDH can also be used as a biocatalyst in a fusion state capable of stably converting a substrate for a long time using these techniques. Additionally, it would be interesting to test the fusion ability of monomeric IDH with other cytochrome P450 and related NADPH-dependent enzymes such as Baeyer-Villiger monooxygenase [14].

**Figure 4:**
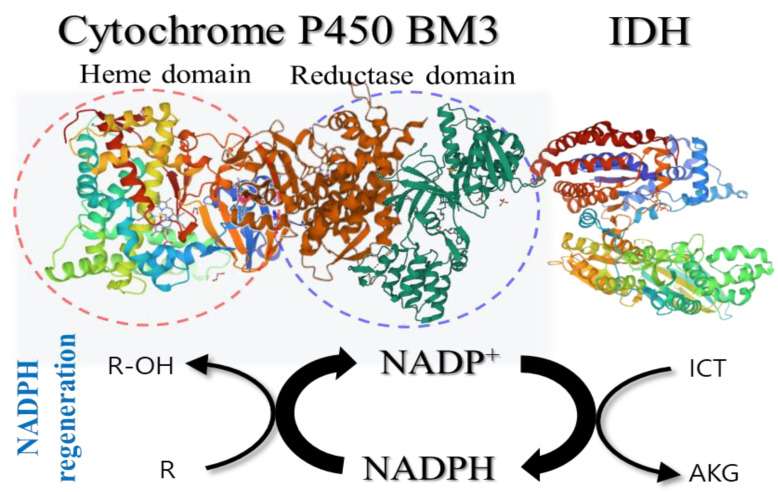
Schematic representation of the coupling reaction between cytochrome P450 BM3 and IDH for NADPH regeneration in a single polypeptide. The structure of the IDH from the *Corynebacterium glutamicum* and two domains of P450 BM3 was obtained from Protein Data Bank (PDB). R, substrate; R-OH, hydroxylated substrate; ICT, isocitrate; AKG, α-ketoglutarate.

Taken together, our findings indicated that monomeric IDH could be used as a viable alternative to replace the enzymes used in the existing NADPH-regeneration system and is expected to contribute to the development of self-sufficient biocatalysts in the form of a fusion protein, which can be more easily implemented compared to conventional multimeric enzymes (Figure 4). Although the activity and stability of the proposed fusion proteins require further improvement, our study provide valuable information on the application of monomeric recombinant IDH to NADPH-regeneration systems. Thus, we expect that monomeric IDH-based NADPH-regeneration systems, especially in the form of a self-sufficient fusion protein, could become the basis for the development of low-cost and high-efficiency biocatalysts.

## 4. Materials and Methods

### 4.1. Bacterial Strains, Plasmids, and Cloning

*E. coli* XL1-Blue *(recA1 endA1 gyrA96 thi-1 hsdR17 supE44 relA1 lac* [F’ *proAB lacI^q^ZΔM15* Tn10 (Tet^r^)]) was used as a host to construct the recombinant plasmid and *E. coli* BL21 (DE3) (F*^−^ ompT hsdS_β_(r_β_^−^m^−^) dcm gal* λ (DE3)) was used as a host for recombinant protein expression. The whole nucleotide sequences encoding a monomeric IDH from *C. glutamicum* (CgIDH, Gene ID: X71489) and *A. vinelandii* (AvIDH, Gene ID: D73443) and dimeric G6PDH from *L. pseudomesenteroids* (LmG6PDH, Gene ID: M64446) were obtained from ENA of the EMBL-EBI. The full-length genes were amplified from genomic DNA by PCR with specifically designed primers (BIONICS, Daejeon, South Korea) with a 6xHis tag at the C-terminal region. The recombinant plasmid pCW-BM3, containing a gene encoding cytochrome P450 BM3 M10, was used as the template to amplify the BM3 gene by PCR with a set of specific primers [28]. The PCR products were purified and subcloned into the pET24a expression vector by homologous recombination using an In-Fusion HD cloning kit (Takara Bio USA, San Jose, CA, USA). Each cloned gene was confirmed by colony PCR, followed by DNA sequencing (BIONICS, Daejeon, Republic of Korea). The plasmids and primers used in this study are listed in Table 3.

### 4.2. Protein Expression and Purification

Each recombinant plasmid harboring the gene encoding CgIDH or AvIDH was transformed into *E. coli* BL21(DE3) using the heat shock method and then spread on Luria-Bertani (LB) solid media. The resulting single colony was randomly selected and seeded into 3.5 mL of LB medium containing 50 μg/mL kanamycin, then further grown at 37 °C under constant shaking (200 rpm). After cultivation for 4 h, 2% (*v*/*v*) of the resulting culture was inoculated into 150 mL of LB medium containing 50 μg/mL kanamycin and incubated under the same conditions. Upon reaching an optical density of approximately 0.6–0.8 at 600 nm (OD_600_), 0.2 mM isopropyl β-D-1-thiogalactopyranoside (IPTG) was added to the culture medium to induce protein expression for 3 h at 30 °Cunder constant shaking (200 rpm). The cultivated cells were harvested by centrifugation at 6000× *g* for 10 min and resuspended in 40 mL of phosphate-buffered saline (PBS, pH 7.4), then sonicated for 2 min (40% amplified, 2 s ON, and 8 s OFF) at 4 °C. The insoluble aggregate in the lysate was removed by centrifugation at 16,100× *g* for 1 h at 4 °C. Subsequent purification steps were conducted as described in a previous report with slight modifications [32]. The resulting supernatant was applied to a 5 mL HisTrap crude FF column (Cytiva, Marlborough, MA, USA) that was pre-equilibrated with PBS (pH 7.4) on a fast protein liquid chromatography system (AKTA purifier, GE Healthcare, Chicago, IL, USA). After binding, the column was thoroughly washed with PBS (50 bed volumes) containing 10 mM imidazole and then eluted with a linear gradient of up to 100% elution buffer (250 mM imidazole) within 10 column volumes (CV). The fractions containing the protein of interest were analyzed via SDS-PAGE (10%) and subjected to densitometry scanning for the determination of purity. The eluted enzyme was desalted and concentrated by ultrafiltration using a Centricon centrifugal filter (Millipore, Burlington, MA, USA). The concentration of the purified recombinant protein was determined via the Bradford assay using bovine serum albumin (BSA) as a standard.

### 4.3. Size Exclusion Chromatography

Analytical Size exclusion chromatography (SEC) was performed using Superdex 200 Increase 10/300 GL column (Cytiva, USA) equilibrated with PBS (pH 7.4). The purified recombinant proteins (75–100 μg) were injected into the column and the analyses were conducted at a fixed flow rate of 0.5 mL/min. The elution profile was monitored at wavelength of 280 nm. The column was calibrated using size markers as follows: thyroglobulin (669 kDa), ferritin (440 kDa), aldolase (158 kDa), conalbumin (75 kDa), ovalbumin (44 kDa) carbonic anhydrase (29 kDa), and ribonuclease A (13.7 kDa).

### 4.4. Enzyme Assay and Kinetic Parameters

Recombinant IDH activity assays were performed at 30 °C in a 96-well microplate format with a final volume of 100 μL per well. The assay components including 0.5 mM NADP^+^, 5 nM monomeric IDH, 1 mM Mn^2+^, and 1 mM DL-isocitrate were mixed in 100 mM potassium phosphate buffer (KPi, pH 7.4). Changes in NADPH fluorescence in the reaction solution were monitored at an excitation wavelength of 375 nm and an emission wavelength of 450 nm using a fluorometer (Infinite M200, TECAN, Männedorf, Switzerland). All assays were conducted in triplicate. One unit of enzyme activity was defined as the amount of enzyme required to reduce 1 μmol NADPH per min under the specified conditions. To determine the kinetic parameters for the substrate DL-isocitrate, the initial reaction velocity of monomeric IDH (5 nM) was measured at 30 °C in a 100 μL volume containing 20 mM MOPS (pH 7.4), 100 mM NaCl, 1 mM MnCl_2,_ and 1 mM NADP^+^. The reaction was evaluated different substrate concentrations from 0 to 100 μM under the defined reaction conditions. To measure the kinetic constants for NADP^+^, the isocitrate concentration was maintained at 1 mM with varying cofactor concentrations. The kinetic parameters were calculated from the Michaelis-Menten model using the GraphPad Prism 8.0 (GraphPad Software, San Diego, CA, USA). Optical temperature and pH were determined as previously reported [33]. The effects of varying concentrations of metal ions and salts (i.e., typical additives in enzymatic reactions) on enzyme activity were also analyzed as reported in a previous study [34].

### 4.5. Coupling Reactions of IDH with NADPH-Dependent P450 BM3

The coupling reaction between cytochrome P450 BM3 M10 and monomeric IDH for NADPH regeneration was conducted as previously reported with slight modifications [35]. The coupling reactions of BM3 M10 with IDHs were performed at 37 °C in 100 mM KPi (pH 7.4) buffer containing 200 nM BM3 M10, 1 mM omeprazole (OMP), 1% DMSO, 0.5 mM NADP^+^, 5 nM IDH, 1 mM Mn^2+^, and 1 mM DL-isocitrate. Changes in NADPH fluorescence in the reaction mixture were monitored using an Infinite M200 microplate reader. To quantify the hydroxylation product of OMP obtained through the activity of BM3 M10, samples in which the enzymatic reaction was stopped by adding cold methanol (1:1) were analyzed using a high-performance liquid chromatography system (Alliance e2695, Waters, Milford, MA, USA) as reported previously [28,35].

## Figures and Tables

**Table 1:** Kinetic parameters, optimum temperature and pH of recombinant CgIDH and AvIDH.

Enzymes	NADP^+^			Isocitrate			Ref.
	*K*_m_ (μM) *	*k*_cat_ (s^−1^)	*k*_cat_/*K*_m_ (μM^−1^s^−1^)	*K*_m_ (μM)	*k*_cat_ (s^−1^)	*k*_cat_/*K*_m_ (μM^−1^s^−1^)	
Previously reported CgIDH	4.0	87.0	21.8	5.0	87.0	17.4	[25]
Previously reported AvIDH	5.8	92.6	15.9	7.9	73.0	9.3	[26]
CgIDH	10.3	43.7	4.3	15.0	49.4	3.3	This study
AvIDH	5.9	38.7	6.6	13.9	44.0	3.2	This study
**Enzyme**	**Optimum Temperature (°C)**			**Optimum pH**			
CgIDH	40			7.5			
AvIDH	40			7.5			

* The kinetic parameters *K*_m_ and *k*_cat_ were calculated via nonlinear least squares fitting of the data to the Michaelis-Menten equations for NADP^+^ and isocitrate. The initial reaction velocities of both enzymes were determined as described in the Materials and Methods. The reaction products were analyzed by using either spectrofluorometric or the HPLC method as described in Materials and Methods. Each value represents the average of three independent assays.

**Table 2:** Metal dependency of recombinant CgIDH and AvIDH.

Metal Ions *	Relative Activity (%)	
	CgIDH	AvIDH
EDTA	ND	ND
Mn^2+^	100 ± 6.1	100 ± 6.0
Mg^2+^	43.7 ± 2.9	50.0 ± 3.5
Ca^2+^	2.1 ± 0.3	1.3 ± 0.2
Cu^2+^	2.6 ± 0.5	2.1 ± 0.2
Co^2+^	6.4 ± 0.9	7.8 ± 0.3
Ni^2+^	2.7 ± 0.2	2.2 ± 0.1
Zn^2+^	8.8 ± 0.8	11.7 ± 0.1

* Preincubation with metal ions was carried out at 30 °C for 10 min. Each value represents the average of three independent assays. ND means not detected.

**Table 3:** Plasmids and primers used in this study.

Names	Content & Sequence	RE *
Plasmids		
pET24a	Kan^r^, T7 promoter, *lacO*, PBR322 ori	
pET24a-CgIDH	*idh* gene from *C. glutamicum* subcloned into pET24a	
pET24a-AvIDH	*idh* gene from *A. vinelandii* subcloned into pET24a	
pET24a-LmG6PDH	*g6pdh* gene from *L. mesenteroides* subcloned into pET24a	
pET24a-BM3	*CYP102A1* gene from *B. megaterium* subcloned into pET24a	
Primers		
CgIDH F	5′-GAAGGAGATATACATATGGCTAAGATCATCTGGACCCG-3′	*Nde*I
CgIDH R	5′-GTGGTGGTGGTGCTCGAGCTTCTTCAGTGCGTCAACGATCTC-3′	*Xho*I
AvIDH F	5′-GAAGGAGATATACATATGTCCACACCGAAGATTATCTATACGC-3′	*Nde*I
AvIDH R	5′-GTGGTGGTGGTGCTCGAGTGCAAGAGGTGCCAGAGCC-3′	*Xho*I
LmG6PDH F	5′-TAAGAAGGAGATATACATATGGTTTCAGAAATCAAGACGTTAGTAACTTTC-3′	*Nde*I
LmG6PDH R	5′-GTGGTGGTGGTGCTCGAGACCTTTAAACACCCAAGCATCACC-3′	*Xho*I
BM3 F	5′-TAACAATTCCCCTCTAGAAATAATTTTGTTTAACTTTAAGAAGGA GATATACATATGAC-3′	*Xba*I
BM3 R	5′-GCTGCCTCCTTTGCTGCTGCCTCCTTAGCAGCAGCTTCCCCAGCCC ACACGTCTTTTGC-3′	

* Restriction enzymes used to digest the amplified DNA fragment by PCR and the target sequences incorporated in primer for the digestion with restriction enzymes are underlined.

## Data Availability

Not applicable.

## Supplementary Materials

The following supporting information can be downloaded at: https://www.mdpi.com/article/10.3390/ijms232315318/s1.
