# Supplementary material for: Expression and Characterization of Monomeric Recombinant Isocitrate Dehydrogenases from Corynebacterium glutamicum and Azotobacter vinelandii for NADPH Regeneration
Source: Int J Mol Sci. 2022 Dec 5;23(23):15318. doi: 10.3390/ijms232315318 (PMC9736777; doi:10.3390/ijms232315318)
Supplement: Supplementary file 1 [file ijms-23-15318-s001.zip › ijms-2028651-supplementary.pdf]

Supplementary Materials

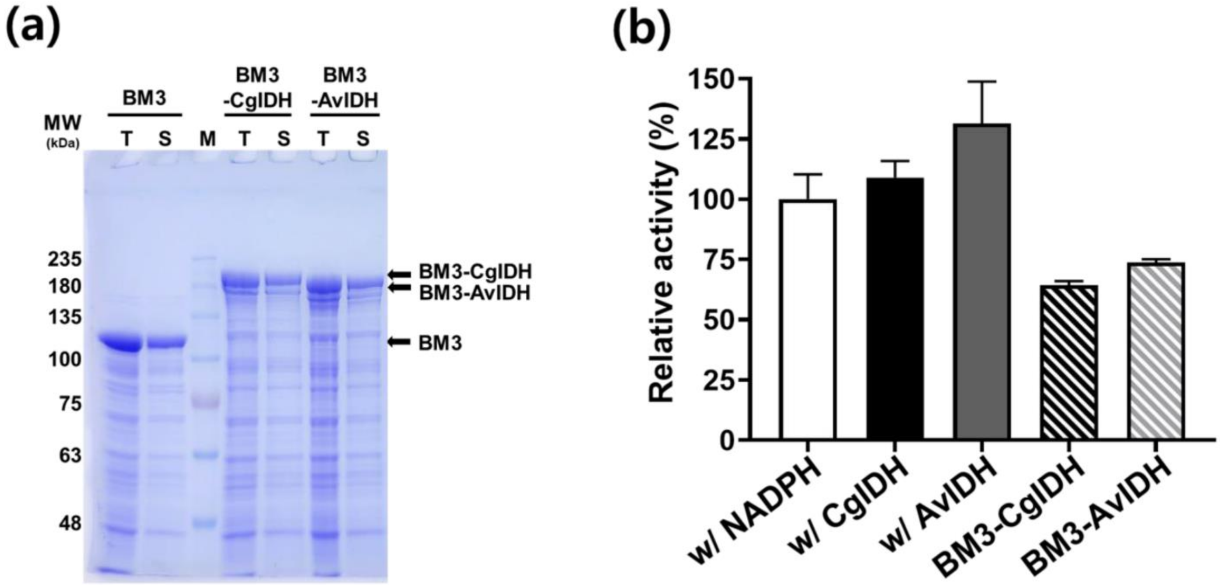

Figure S1. Amino acid sequence alignment of the two isocitrate dehydrogenase from *C. glutamicum* (CgIDH) and *A. vinelandii* (AvIDH). Identical and conservative substitutive amino acid residues are indicated in blue and sky blue boxes, respectively.

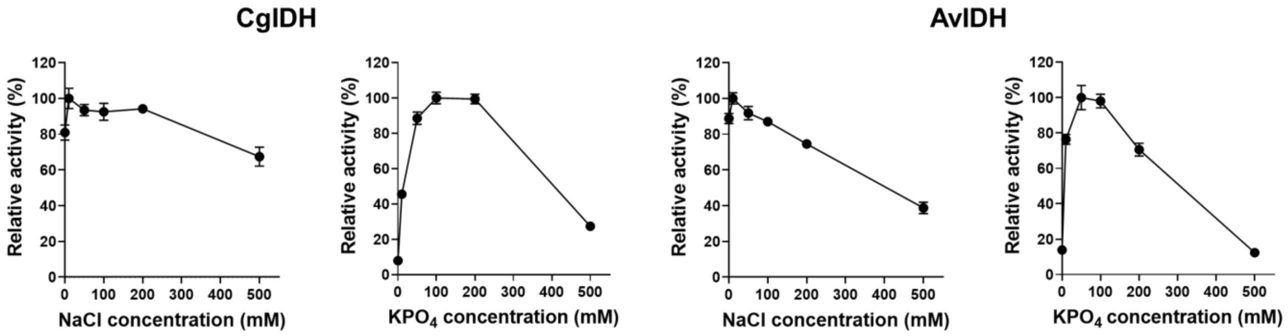

Figure S2. Effects of salt concentration on enzyme activity of IDHs. The effects of salts (NaCl and KPi) on enzyme activity were determined by a typical assay method (as described in the Materials and Methods) using a buffer containing different concentrations of NaCl and KPi. The relative activity was calculated by defining the highest activity of the sample as 100%. The values are expressed as means ± SDs of three independent experiments.

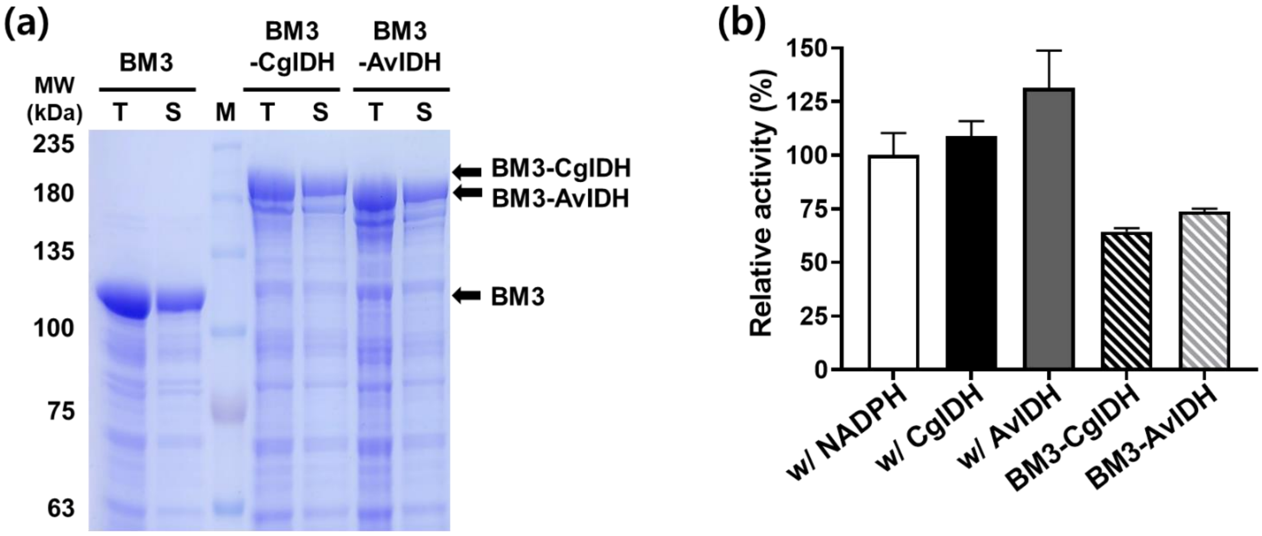

Figure S3. Analyses of expression level and coupling activity of fusion proteins BM3-CgIDH and BM3-AvIDH. (a) SDS-PAGE analyses of the expressed fusion proteins BM3-IDHs. Aliquots of protein samples were analyzed on an 8% PAGE gel under denaturing conditions. The expression level and solubility of recombinant BM3-CgIDH and BM3-AvIDH were analyzed under the same induction conditions at 30℃. M, molecular weight size marker; T, total protein fraction; S, soluble protein fraction. The arrow indicates the bands corresponding to the recombinant fusion protein. (b) Coupling reaction of the two fusion proteins BM3-CgIDH and BM3-AvIDH for NADPH regeneration in a single polypeptide. The coupling reactions were carried out following the same procedure as in Figure 3. Aliquots of coupled reaction samples were taken after 30 min of reaction and treated with cold-methanol (1:1), then analyzed by high-performance liquid chromatography. As the positive control, the reaction supplemented with independent (not fused) enzymes was carried out under the same conditions. A BM3 reaction with NADPH instead of $NADP^+$ was also used as a control.
